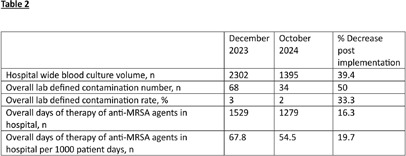# Impact of Electronic Health Record Embedded Guidance on Contaminated Blood Cultures and Anti-MRSA Agent Utilization

**DOI:** 10.1017/ash.2025.279

**Published:** 2025-09-24

**Authors:** Jordan Chiasson, Michael Kent, Michelle Galvez, Jeremie Sawadogo, Rajasekhar Jagarlamudi

**Affiliations:** 1 JPS Health Network; 2 JPS Health Network; 3 John Peter Smith Hospital; 4 JPS Health Network; 5 JPS Health System

## Abstract

**Background:** Blood culture contamination is a large burden on the health system with significant excessive costs and antimicrobial use. In 2024, a national blood culture shortage required intensive conservation strategies regarding blood culture collection. We developed clinical guidance on blood culture utilization and embedded it in electronic health record (EHR). Our goal is to evaluate its impact on blood culture utilization and anti-MRSA agent usage at our institution. **Methods:** The antimicrobial stewardship team provided educational communication, and blood culture bottle conservation strategy (BCBCS) recommendations that were embedded into the EHR in July 2024 (Figure 1). Patient charts with a laboratory identified blood culture growing a contaminant in December 2023 (prior to BCBCS) and October 2024 (post-BCBCS) were reviewed. Patients were excluded if they had another clinically relevant pathogen in blood cultures, were discharged prior to blood culture result, or died within 48 hours of blood culture result. Information on anti-MRSA agent (vancomycin, linezolid, daptomycin, ceftaroline) days of therapy (DOT), total hospital blood culture volume, blood culture contamination rates, and ID consultation was collected. **Results:** 54 patients pre-BCBCS and 29 patients post-BCBCS were reviewed. Anti-MRSA DOT in patients reviewed with contaminated cultures was 161 pre-BCBS and 56 post-BCBCS (Table1). Overall blood culture volume and contamination rate were reduced post BCBCS implementation (Table 2). Total hospital anti-MRSA DOT was noted to be less post EHR guidance as well (1529 pre-BCBCS and 1279 post-BCBS). **Conclusions:** Reduction in both the volume of blood culture collection and overall contamination rate contributed to a reduction of anti-MRSA therapy at our institution. These results highlight the impact that diagnostic stewardship may have on antimicrobial stewardship metrics.

**Figure:**
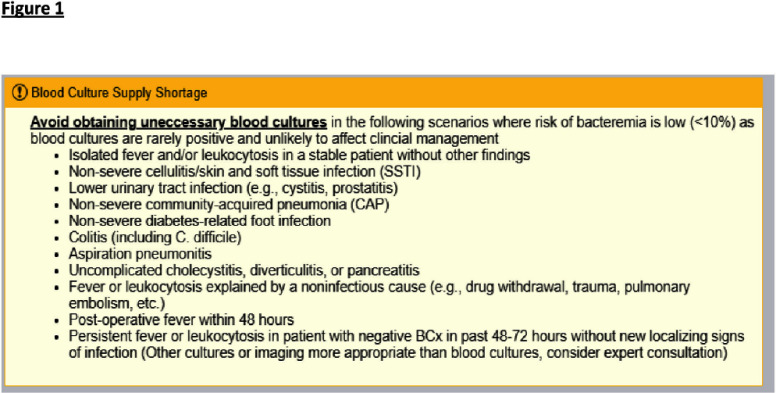

**Figure:**
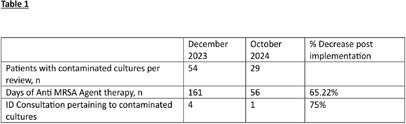

**Figure:**